# Testosterone and Cortisol Responses to Five High-Intensity Functional Training Competition Workouts in Recreationally Active Adults

**DOI:** 10.3390/sports6030062

**Published:** 2018-07-14

**Authors:** Gerald T. Mangine, Trisha A. Van Dusseldorp, Yuri Feito, Alyssa J. Holmes, Paul R. Serafini, Allyson G. Box, Adam M. Gonzalez

**Affiliations:** 1 Department of Exercise Science and Sport Management, Kennesaw State University, Kennesaw, GA 30144, USA; tvanduss@kennesaw.edu (T.A.V.D.); yfeito@kennesaw.edu (Y.F.); aholme35@students.kennesaw.edu (A.J.H.); pserafi1@students.kennesaw.edu (P.R.S.); agbox2@illinois.edu (A.G.B.); 2 Department of Health Professions, Hofstra University, Hempstead, NY 11549, USA; adam.m.gonzalez@hofstra.edu

**Keywords:** salivary steroid, acute endocrine response, CrossFit^®^, testosterone-to-cortisol ratio

## Abstract

To determine the salivary steroid response to high-intensity functional training (HIFT) competition workouts, saliva samples were collected from ten recreationally trained male and female competitors during a 5-week (WK1–WK5) international competition. Competitors arrived at their local affiliate and provided samples prior to (PRE) their warm-up, immediately (IP), 30-min (30P), and 60-min (60P) post-exercise. Samples were analyzed for concentrations of testosterone (T), cortisol (C), and their ratio (TC). Generalized linear mixed models with repeated measures revealed significant main effects for time (*p* < 0.001) for T, C, and TC. Compared to PRE-concentrations, elevated (*p* < 0.05) T was observed at IP on WK2–WK5 (mean difference: 135–511 pg·mL^−1^), at 30P on WK3 (mean difference: 81.0 ± 30.1 pg·mL^−1^) and WK5 (mean difference: 56.6 ± 22.7 pg·mL^−1^), and at 60P on WK3 (mean difference: 73.5 ± 29.7 pg·mL^−1^) and WK5 (mean difference: 74.3 ± 28.4 pg·mL^−1^). Compared to PRE-concentrations, elevated (*p* < 0.05) C was noted on all weeks at IP (mean difference: 9.3–15.9 ng·mL^−1^) and 30P (mean difference: 6.0–19.9 ng·mL^−1^); significant (*p* < 0.006) elevations were noted at 60P on WK1 (mean difference: 9.1 ± 3.0 ng·mL^−1^) and WK5 (mean difference: 12.8 ± 2.9 ng·mL^−1^). Additionally, TC was significantly reduced from PRE-values by 61% on WK1 at 60P (*p* = 0.040) and by 80% on WK5 at 30P (*p* = 0.023). Differences in T, C, and TC were also observed between weeks at specific time points. Although each workout affected concentrations in T, C, and/or the TC ratio, changes appeared to be modulated by the presence of overload and workout duration. During periods of elevated training or competition, athletes and coaches may consider monitoring these hormones for consistency and as a means of assessing workout difficulty.

## 1. Introduction

High-intensity functional training (HIFT) is a group-based fitness strategy that utilizes aerobic, gymnastic, and weightlifting exercises performed at a high-intensity and in a constantly varied fashion to promote general physical preparedness [1]. Training sessions will prescribe one or more exercises at a standard volume load that must be completed as fast as possible or for “as many repetitions as possible” (AMRAP) within a given time domain [1]. Though less common, workouts may also require the individual to determine their maximal strength in an exercise within a specified repetition range (i.e., ≥one-repetition maximum) and time. The popularity of this training strategy (e.g., CrossFit^®^) has grown exponentially. Since 2005, the number of CrossFit^®^ affiliates has increased ~1000 fold (from 13 to >13,000) and participation in the CrossFit^®^ Open (CFO) has increased from 26,000 competitors in 2011 to over 380,000 competitors in 2017 [2,3]. Despite its rapid growth and popularity, little is known about the physiological responses to HIFT.

Limited data suggests that HIFT is indeed a high-intensity (or vigorous) exercise strategy. Although the severity differs between workout designs [4], they have consistently been observed to increase heart rate beyond 85% of maximum [5,6,7,8] with elevated concentrations of blood lactate (8–12 mmol·L^−1^) [4,6], markers of oxidative stress [5], and inflammation [7]. Despite these noted responses, little is known regarding the acute hormonal response to this exercise strategy. Kliszczewicz and colleagues (2016) reported a greater catecholamine response to a common HIFT workout compared to time-equated treadmill running (target heart rate = 90% of maximum) for up to one-hour post-exercise. However, differences in exercise modality and intensity (i.e., exercising heart rate was higher during the HIFT workout) make it difficult to make definitive conclusions. Nevertheless, the observed catecholamine responses resemble those of high-volume, short rest interval resistance training programs [9,10,11] and high-intensity interval training [10]. It is possible that other hormones known to respond to high-intensity exercise (i.e., testosterone and cortisol) [9,10,11] may be augmented following various HIFT workouts.

Testosterone (T) is an anabolic hormone that leads to the activation of many important anabolic processes including increases in transcription, translation, signaling-enzymes, and structural proteins [12]. While the physiological significance of its response to exercise has yet to be determined, transient elevations in T may be important for strength adaptations and hypertrophy [12,13,14], as well as the psychological preparedness of an athlete for competition [15]. Aside from its known influence on protein synthesis within the muscle cell, T may also affect cellular processes in a manner that is independent of its receptor [12]. In this capacity, T has been reported to stimulate transient increases in intracellular calcium [16], which may temporarily elevate maximal force production [13] and enable the utilization of greater training intensity and volume-loads. Additionally, elevations in T have been positively associated with competitive anxiety/anticipation, motivation, and aggression (i.e., a “desire to win”) prior to and during a competitive event, all of which may also affect the post-exercise response, depending on the outcome [15]. Conversely, cortisol (C) is a catabolic hormone that responds to psychological and physiological stressors to mobilize energy lipolysis and proteolysis [17]. Although transient, elevations in T and C are expected to occur during and immediately following intense exercise and competition [9,10,11,15], their cumulative effect is not well understood. It has been suggested that the parallel elevations observed in T and C surrounding competition are the result of the adrenal cortex and gonads responding similarly to the physical and psychological elements of competition [15]. Nevertheless, their interaction with hypothalamic-pituitary-adrenal axis [18] and potential competition within the cell nucleus to exert their respective effects [19,20] are of interest. Elevated concentrations of C can inhibit the anabolic effects of T, and thus, the ratio between testosterone and cortisol (TC) concentrations may affect performance and potential training adaptations. 

Evidence suggests that several programming variable combinations will influence the acute hormonal response to resistance exercise [12,17,21]. Typically, however, workouts that elicit a greater metabolic demand (i.e., produce significant elevations in blood lactate) will produce a greater C response [9,10,11,22], whereas most resistance training protocols that overload skeletal muscle will evoke elevations in T [11,12,22,23]. Within an athletic context, a parallel increase in T and C may be expected during the period of competition [15]. The CFO is a five-week, five-workout competition that represents the preliminary stage of the Reebok CrossFit^®^ Games^TM^. Although the format and composition for each CFO workout is unique, they typically resemble HIFT workouts [1]; they are programmed at higher intensities and/or repetition volumes with rest intervals occurring at the competitor’s discretion. Additionally, competitors have the option of completing the workouts ‘as prescribed’ (Rx) or a scaled-down version that adjusts intensity and skill requirements, though it may be assumed that maximal effort is present regardless of the workout’s version when it occurs during competition. However, no study has examined the endocrine response to CFO workouts in any population. Thus, the purpose of the present study was to describe and compare the T and C responses to each week of the CFO competition in recreationally trained adults (i.e., the typical HIFT practitioner). We hypothesized that each CFO workout would elicit robust elevations in T and C. However, because each workout would be performed within the same competitive setting and with maximal effort, the T and C responses would not be different between weeks.

## 2. Materials and Methods

### 2.1. Participants

Five, male (34.4 ± 3.8 years; 175.5 ± 5.1 cm; 80.3 ± 9.7 kg) and five, female (35.5 ± 7.0 years; 159.0 ± 7.1 cm; 76.9 ± 21.4 kg) recreationally trained HIFT practitioners who were enrolled to compete in the CFO were recruited for this study. Following an explanation of all procedures, risks and benefits, each participant provided his/her written informed consent to participate. This investigation was approved by the University’s Institutional Review Board. All participants were free of any physical limitations (determined by medical history questionnaire and PAR-Q). Participants were identified as being “recreationally trained” because they had been regularly participating (at the time of recruitment) in CrossFit^®^ for a minimum of 2 years, and they had never progressed beyond the preliminary stage of the Reebok CrossFit Games^TM^ (i.e., the CFO). Of the five females included in this study, four were naturally cycling while the fifth was using a hormone-based contraceptive (Medroxyprogesterone). However, because the post-exercise response in T and C is not expected to differ between women who use hormone-based contraceptives and those who do not [24,25], this participant’s data was included in our analyses. During the 5-week competition (WK1–WK5), two male competitors missed WK5 due to an illness (*n* = 1) and personal reasons (*n* = 1). Another male participant could not supply a sufficient saliva sample on WK1.

### 2.2. Study Design

Saliva samples were collected prior to and following exercise on each week of the competition. During the first four weeks (WK1–WK4), data for all competition workouts were collected at mid-day (12:00–3:00 p.m. EST (Eastern Standard Time)). The same four participants began these workouts on each Friday at ~2:00 p.m. EST, while the remaining participants always began these workouts on each Saturday at ~12:00 p.m. EST. The final competition workout (WK5) was initiated by all participants at night, within 30 min of its release (~8:00 p.m. EST). Saliva samples were analyzed for concentrations of T and C.

### 2.3. Online Fitness Competition

Competition officials released CFO workouts individually on each Thursday (5:00 p.m. pacific time) of the 5-week competition and competitors could complete multiple attempts before submitting their best attempt by the following Monday (5:00 p.m. pacific time). Each competition workout consisted of two or more exercises performed for a prescribed (Rx) number of repetitions using standardized technique and/or resistance. A “Scaled” option, which consisted of modified technical requirements and/or reduced resistance, was also available. Regardless, competition workouts were scored by the number of repetitions completed within a set time frame (WK1–WK4) or by time to completion (TTC; WK5). All competition workout descriptions, standards, and scoring criteria are available on the competition website [26] and are summarized in Table 1. For the present study, participants were asked to select the competition workout option (Rx or Scaled) that would allow them to “perform their best”, and only their first attempt was considered to minimize the potential influence of an unfamiliar setting, fatigue, or workout familiarity on the endocrine response. All performances were analyzed together due to the assumption that maximal effort was given by all participants for each competition workout. All competition workouts were completed at the same CrossFit^®^ affiliate in front of an official judge.

### 2.4. Saliva Sample Collection and Analysis

Saliva samples were obtained prior to exercise (PRE), immediately post-exercise (IP), 30 min post-exercise (30P), and 60 min post-exercise (60P). Competitors reported to the CrossFit^®^ affiliate approximately 30–40 min before attempting the competition workout and having refrained from consuming any food, drinking hot fluids, or brushing their teeth for two hours prior to their arrival. Upon their arrival, participants were asked to remain seated for 15 min before providing their resting sample (i.e., the PRE-sample). Subsequently, all competitors completed a self-selected warm-up for approximately 10–15 min before attempting the competition workout. IP saliva samples were taken within one minute of workout completion. Participants were then asked to remain in a relaxed position (e.g., seating or standing) while at the affiliate leading up to the 30P and 60P collection time points. The participants could drink water ad libitum while waiting but were asked to refrain from drinking water within 10 min of any post-exercise collection time point.

Approximately 2 mL of saliva were collected into a cryovial (Salimetrics LLC, State College, PA, USA) in duplicate at each time point using the passive drool method and stored at −80 °C until assay. Concentrations of T and C were assessed via commercially available, enzyme-linked immunosorbent assays (ELISA) and a spectrophotometer (SpectraMax M3, Molecular Devices, Sunnyvale, CA, USA). To eliminate inter-assay variance, all samples were thawed once and analyzed in duplicate in the same assay run by a single technician, with an average coefficient of variation (CV) of 9.92% for T and 9.76% for C.

### 2.5. Statistical Analyses

Changes in concentrations of T, C, and the TC ratio were separately examined across time using a generalized linear mixed model with maximum likelihood estimation and an autoregressive-heterogenous repeated covariance to account for the dependent relationships existing between time points. Due to known effects of sex on steroid hormone concentrations [27,28], sex was added as a factor into the model to test for differences. Following a significant F-ratio, specific differences were further assessed by applying adjustments to confidence intervals using the least significant difference procedure. All data are reported as mean ± standard error. SPSS statistical software (SPSS, v.24, Chicago, IL, USA) was used for all analyses with statistical significance set at *p* < 0.05.

## 3. Results

### 3.1. Online Fitness Competition

Throughout the CFO, participants did not report any changes to their normal dietary or training habits. The number of participants completing each workout version (Rx or Scaled) varied each week. Specifically, the number of female competitors who used Rx standards varied between WK1 (*n* = 1), WK2 (*n* = 3), WK3 (*n* = 2), WK4 (*n* = 1), and WK5 (*n* = 4). In contrast, all male participants completed all workouts using Rx standards. The selected workout format and performance scores for each competitor across each week of the CFO are presented in Table 2.

### 3.2. Testosterone

**Figure 1:**
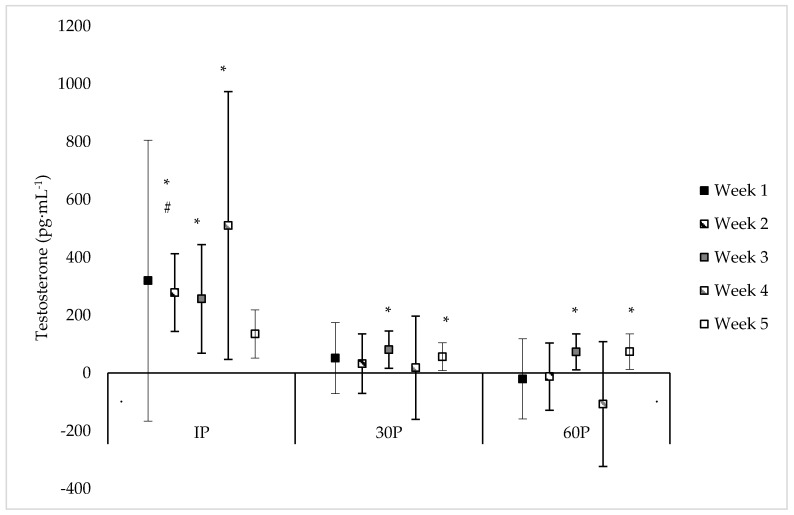
Mean changes in testosterone concentrations during each week of the CrossFit^®^ Open (mean difference ±95% Confidence Interval). * = Significantly (*p* < 0.05) different from PRE-scores; **#** = Significantly (*p* < 0.05) different from WK5.

Neither a significant sex x time (F = 1.18, *p* = 0.318) or main effect for sex (F = 0.01, *p* = 0.923) were observed for T concentrations. However, a significant main effect for time was observed (F = 4.01, *p* < 0.001) where T concentrations were elevated from PRE-values at IP on WK2–WK5 (mean difference: 135–511 pg·mL^−1^, *p* ≤ 0.033), at 30P on WK3 (mean difference: 81.0 ± 30.1 pg·mL^−1^, *p* = 0.017) and WK5 (mean difference: 56.6 ± 22.7 pg·mL^−1^, *p* = 0.024), and at 60P on WK3 (mean difference: 73.5 ± 29.7 pg·mL^−1^, *p* = 0.023) and WK5 (mean difference: 74.3 ± 28.4 pg·mL^−1^, *p* = 0.021). On specific time points between weeks, T concentrations were significantly higher (*p* = 0.036) at IP on WK2 (487 ± 113 pg·mL^−1^) compared to WK5 (229 ± 49 pg·mL^−1^), and significantly higher (*p* = 0.023) at 30P on WK4 (318 ± 79 pg·mL^−1^) compared to WK5 (150 ± 29 pg·mL^−1^). Additionally, compared to WK5, trends were noted for higher T concentrations at PRE on WK3 (mean difference: 35.8 ± 19.1 pg·mL^−1^, *p* = 0.071), at IP on WK4 (mean difference: 580 ± 295 pg·mL^−1^, *p* = 0.064), and at 30P on WK2 (mean difference: 91.3 ± 52.2 pg·mL^−1^, *p* = 0.090). No other differences were found in T concentrations within or between each week. Individual values for T concentrations across each time point are presented in Table 3. Differences between post-exercise T concentrations and PRE-concentrations during each week of the CFO, as well as differences between weeks at specific time points, are illustrated in Figure 1.

### 3.3. Cortisol

**Figure 2:**
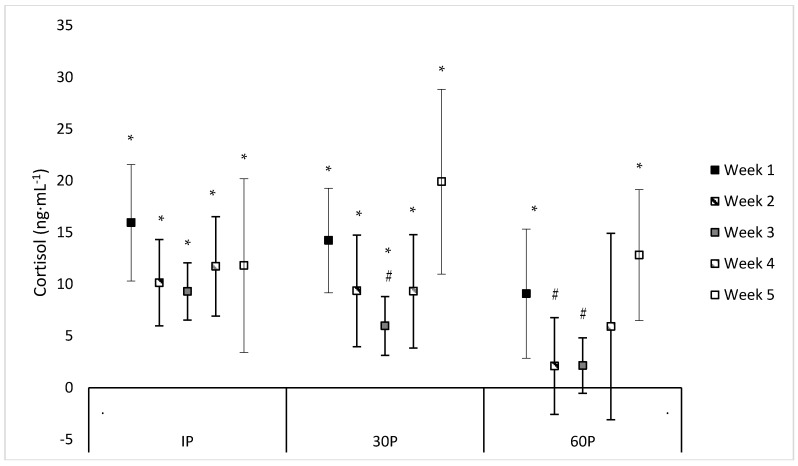
Mean percent changes in cortisol concentrations during each week of the CrossFit^®^ Open (mean difference ±95% Confidence Interval). * = Significantly (*p* < 0.05) different from PRE-scores. # = Significantly (*p* < 0.05) different from WK1.

Neither a significant sex x time (F = 1.07, *p* = 0.414) or main effect for sex (F = 0.96, *p* = 0.340) were observed for C concentrations. However, a significant main effect for time was observed (F = 10.13, *p* < 0.001) where C concentrations were elevated from PRE-values at IP on WK1–WK5 (mean difference: 9.3–15.9 ng·mL^−1^, *p* ≤ 0.011), at 30P on WK1–WK5 (mean difference: 6.0–19.9 ng·mL^−1^, *p* ≤ 0.002), and at 60P on WK1 (mean difference: 9.1 ± 3.011 ng·mL^−1^, *p* = 0.006) and WK5 (mean difference: 12.8 ± 2.9 ng·mL^−1^, *p* < 0.001). Compared to WK5, PRE-concentrations of C were significantly higher (*p* ≤ 0.017) on WK1–WK4 (mean difference: 4.7–7.6 ng·mL^−1^). At 30P, C concentrations were significantly higher (*p* = 0.004) on WK1 (24.1 ± 2.9 ng·mL^−1^) compared to WK3 (14.0 ± 1.7 ng·mL^−1^). Likewise, higher C concentrations were observed at 60P on WK1 (19 ± 3.1 ng·mL^−1^) compared to WK2 (11.8 ± 2.0 ng·mL^−1^) and WK3 (10.2 ± 1.2 ng·mL^−1^). Additionally, a trend was noted for higher C concentrations at IP on WK1 (25.8 ± 3.9 ng·mL^−1^) compared to WK3 (17.4 ± 2.0 ng·mL^−1^, *p* = 0.054). and WK5 (15.1 ± 4.3 ng·mL^−1^, *p* = 0.075), lower C concentrations at 30P on WK3 (14.0 ± 1.7 ng·mL^−1^) compared to WK4 (20.3 ± 3.2 ng·mL^−1^, *p* = 0.055) and WK5 (23.3 ± 4.5 ng·mL^−1^, *p* = 0.062), and lower (*p* = 0.084) C concentrations at 60P on WK3 (10.2 ± 1.2 ng·mL^−1^) compared to WK5 (16.2 ± 3.1 ng·mL^−1^). No other differences were found in C concentrations within or between each week. Individual values for C concentrations across each time point are presented in Table 4. Differences between post-exercise C concentrations and PRE-concentrations during each week of the CFO, as well as differences between weeks at specific time points, are illustrated in Figure 2.

### 3.4. Testosterone-Cortisol Ratio

**Figure 3:**
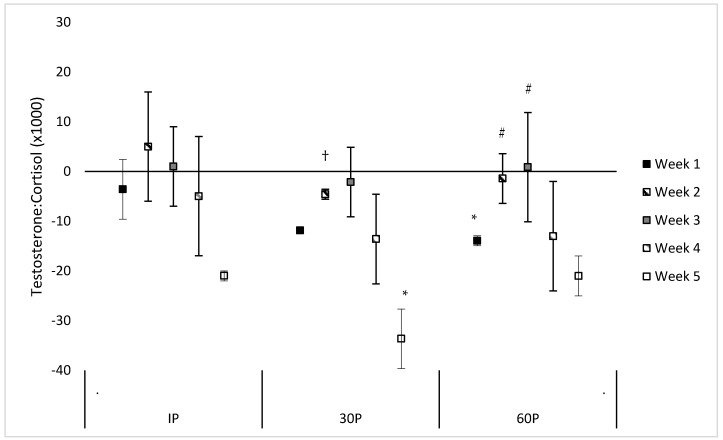
Mean changes in the testosterone-cortisol ratio during each week of the CrossFit^®^ Open (mean difference ±95% Confidence Interval). * = Significantly (*p* < 0.05) different from PRE-scores. # = Significantly (*p* < 0.05) different from WK1. † = Significantly (*p* < 0.05) different from WK5.

Neither a significant sex x time (F = 1.30, *p* = 0.239) or main effect for sex (F = 0.55, *p* = 0.470) were observed for the TC ratio. However, a significant main effect for time was observed (F = 3.56, *p* < 0.001) where the TC ratio was significantly reduced from PRE-values at 30P on WK5 (mean difference: −0.034 ± 0.012, *p* = 0.023) and at 60P on WK1 (mean difference: −0.014 ± 0.006, *p* = 0.040). Trends were also noted for reduced TC ratio on WK1 at 30P (mean difference: −0.012 ± 0.006, *p* = 0.057) and on WK5 at IP (mean difference: −0.021 ± 0.009, *p* = 0.061) and 60P (mean difference: −0.021 ± 0.012, *p* = 0.094). Compared to WK5, a significantly higher TC ratio was observed at 30P on WK2 (mean difference: 0.007 ± 0.003, *p* = 0.038) and trends for a higher TC ratio were observed on WK3 (mean difference: 0.011 ± 0.005, *p* = 0.059) and WK4 (mean difference: 0.011 ± 0.006, *p* = 0.076). Compared to WK1, significantly higher TC ratios were observed at 60P on WK2 (mean difference: 0.009 ± 0.003, *p* = 0.005) and WK3 (mean difference: 0.013 ± 0.004, *p* = 0.002). No other differences were found in the TC ratio within or between each week. Differences between post-exercise TC and PRE-values during each week of the CFO, as well as differences between weeks at specific time points, are illustrated in Figure 3.

## 4. Discussion

The aim of the present study was to describe and compare the acute T and C responses to the five workouts from the 2016 CFO competition. Although CFO workouts are usually novel and vary each year, they can be expected to include movements common to HIFT, set within typical programming schemes (e.g., AMRAP, TTC). Given the consistent variation among each workout, it is not surprising that the available data on this training style also suggests variability in the acute physiological responses of different workouts [6,7]. Fernandez-Fernandez and colleagues (2015) reported differences in the oxygen cost, energy expenditure, and respiratory exchange ratio elicited by two common HIFT workouts. Likewise, differences were noted between two novel, HIFT-style workouts for post-workout concentrations of blood glucose, lactate, and markers of inflammation [7]. In the present study, post-exercise elevations in salivary steroid concentrations were observed on each week, while specific differences between weeks were also noted. Previously, resting T and C concentrations had been tracked over the course of an entire macrocycle in an elite CrossFit^®^ athlete [29]. In that study, T and C concentrations increased from baseline to the competitive period, but their response within the competition itself was not examined. The current investigation appears to be the first to examine these hormones following multiple competitive workouts within the same period in recreational athletes.

As an anabolic hormone, the physiological role of testosterone is to increase protein synthesis through a variety of mechanisms [12], but its role during (and in response to) exercise is not well understood. Preliminary evidence suggests that exercise-induced elevations in T are related to transitory elevations in force producing capabilities of skeletal muscle, potentially hypertrophy [12,13,14], and alterations in mood and aggressiveness [15]. Unfortunately, these hypotheses are difficult to confirm because a variety of exercise modalities and programming designs appear to elicit similar responses [22,23,30]. Among various designs, the most consistent prerequisite for stimulating a T response is “overload” (i.e., challenging the targeted physiological system beyond its norm) [11,12,22,31]. Ahtiainen and colleagues [31] noted greater testosterone concentrations following a bout of forced repetitions compared to finishing sets at failure. While neither HIFT or the CFO typically practice forced repetitions, AMRAP and TTC workouts are likely to present an overload when additional repetitions are completed (or attempted) while fatigued, when participants utilize shorter self-selected rest intervals, or both. 

In the present investigation, significant T elevations were observed on each week except WK1. Though it might be assumed that maximal effort was given on each week, it cannot be confirmed. Therefore, the presence of (or lack of) overload may have been responsible for this inconsistency. Each competitor was given the option of completing the Rx or the scaled version of each workout based on their ability to perform the required exercises and do their best. However, differences between loads prescribed and exercise modifications within each workout version were not based on a specific metric (e.g., percentage of one-repetition maximum or body weight). Rather, the prescribed modifications were standard, which could reduce the available options to being impossible or not sufficiently challenging for individual competitors. An example of this occurred on WK3, where a participant attempted the Rx challenge but could not complete a “muscle-up” which prohibited her from continuing the workout. The lack of a significant T response on WK1 may have been the consequence of athlete workout selection and the differences between Rx and scaled intensities. On WK1, four female competitors chose to complete the scaled version of the workout, which included modified intensity and complexity for each exercise. While four females also chose to complete the scaled version of WK4 and a significant T response was observed, modifications were only made for two of the four prescribed exercises. Comparatively, the scaled version of WK4 also appeared to require more work than the scaled version of WK1. For instance, the first exercise of WK4 required participants to complete 55 deadlift repetitions at 135 lbs. (total volume load = 7425 lbs.) and all participants finished this portion. In contrast, the only exercise on WK1 that required participants to lift external weight was the walking lunge while holding a 35-pound barbell in the front rack position. The female participants, who completed the scaled version of WK1, averaged ~216 repetitions (or 85 walking lunge repetitions) which would roughly equate to a total volume load of 2975 lbs. Had they chosen to complete the Rx version of WK 1 (walking lunges while holding a 65-pound barbell in the overhead position) and completed the same number of repetitions, they would have nearly doubled their total volume load. Though speculative, the increased volume load combined with more difficult body weight exercises (chest-to-bar pull-ups vs. jumping chin-ups and bar-over burpees vs. burpees) may have elicited a more robust T response.

Differences in the T response were also noted between weeks, where elevations in T on WK2 (at IP) and WK4 (at 30P) were ~2.1 times greater than those seen on WK5 at each respective time point. While differences between workouts may have played a role, the reduced sample on WK5 and its occurrence at night prohibit any definitive conclusions. On WK5, two of the male competitors were not available to complete the workouts due to personal reasons. Across all time points, the average T concentrations in these two individuals were approximately 2.4 times greater than those observed in their remaining male counterparts and 1.7 times greater than the combination of all other participants. Previously, greater T concentrations following resistance exercise have been observed in men compared to women [27,28], and the T response in males appears to be more sensitive to psychological factors (e.g., motivation, aggressiveness, and expected performance outcome) known to be present during competition [15,32]. Unfortunately, perception towards the competition was not assessed in this study, and our data did not suggest an effect from sex on the T response; albeit, our overall sample size lacked sufficient power to detect such differences. Nevertheless, the absence of these two participants on WK5 appears to have affected the differences in T concentrations observed between weeks. Another possible explanation could be related to the WK5 workout being completed at night whereas all other challenges were completed during the day. Baseline T concentrations are known to diminish from morning to evening [15] and evidence exists of a diurnal effect on its response to exercise [33]. Thus, it remains unclear whether the observed responses were elicited by each workout’s difficulty, the competition environment, or possibly the competitive nature of the participants [15].

Elevations in C were seen following exercise on each week of the CFO, while specific differences were noted between weeks prior to and following exercise. Prior to exercise, lower C concentrations were observed on WK5 compared to all other weeks. Although it is possible that psychological factors related to stress (e.g., anticipation towards the final competition workout, perception of workout difficulty, attitude towards completing the workout alongside more gym members) may have played a role [15], the clearest difference between WK5 and all other weeks was that it took place at night. Like T, baseline C concentrations are known to diminish from morning to evening [34] and our findings are consistent with that pattern. Following exercise, significantly greater C concentrations were observed on WK1 compared to WK2 (at 60P) and WK3 (at 30P and 60P). Typically, resistance exercise positively affects transitory C concentrations [22], particularly when exercise stimulates an increase in blood lactate concentrations [17,22,31] and when programming incorporates higher training volumes and shorter rest intervals [17]. Here, we did not examine the lactate response and cannot speculate on its relation to elevations in C. Nevertheless, elevated lactate concentrations are common following HIFT-style workouts [4,6] and are modulated by effort [21]. While WK1 may not have provided a sufficient intensity overload to stimulate T, it was the longest (20 min) workout of the CFO, participants completed the most repetitions (~490 repetitions) during this workout, and its AMRAP design encouraged short rest intervals. In contrast, the workout duration for WK2 averaged 9.6 min for our participants and they only averaged 186 repetitions. Likewise, the duration for WK3 was set at 7 min and participants only averaged a total of 77 repetitions. Interestingly, the two “scaled” female participants on WK2 were the only participants to exceed 2 rounds (389 repetitions on average), and their post-exercise C concentrations were approximately 1.7 times greater than all other participants combined. Meanwhile, the longer durations of WK4 (13 min) and WK5 (17.5 min on average), and the inclusion of greater intensity loads, might explain why no significant differences were seen between these weeks and WK1.

At rest, the TC ratio is believed to be indicative of an individual’s current anabolic status because of T and C’s interaction along the hypothalamic-pituitary-adrenal axis [18] and their potential competition within the nucleus [19,20]. Indeed, prolonged elevations in C have been observed to negatively affect skeletal muscle and body mass [20]. However, less is known regarding the importance of the TC ratio following exercise. Previously, Fry et al. [35] observed a decreased TC ratio following an overreaching protocol in elite weightlifters, but not when the workout was repeated after one week of heavy training. Further, the same response pattern was observed after 1-year of training. Using a ≥30% decrease in TC as criteria for overtraining [36], the authors concluded that the consistent response pattern was evidence that the heavy training period was typical of an overreaching phase and not overtraining. In the present study, significant reductions (>30%) in TC only occurred on WK1 (at 60P) and WK5 (at 30P), and appeared to be regulated by the workout’s ability to provide an overload (i.e., stimulate T). For instance, the duration and design of WK1 was sufficient to elicit a response in T, but as we have previously discussed, several participants chose to scale the WK1 which may have limited the T response. Likewise, the design of WK5 was sufficient to stimulate both T and C. However, its duration and lower intensity would appear to have a larger effect on C (444% average post-exercise increase) than T (95% average post-exercise increase). Interestingly, the TC ratio was not affected on WK2 through WK4, and the participants did not report any consistent changes to their normal training regimen. This could imply that the CFO may be considered an overreaching period within the context of normal training in recreationally active HIFT practitioners. Nevertheless, it must be acknowledged that the TC ratio is a volatile measure of anabolic status that can be heavily influenced by changes either T or C [37]. As such, these findings should be considered preliminary and warrant further investigation alongside additional markers of anabolic status and overtraining.

Although HIFT constantly varies the design, intensity, and complexity of workouts, practitioners can modify workouts to best suit their skillset. Within the context of the CFO, these options are limited and may influence the endocrine response to exercise. This presents a challenge when attempting to study responses in recreational competitors (i.e., the typical HIFT practitioner). Given their status, these individuals cannot be expected to be proficient in all possible exercises and/or always able to lift the specific loads prescribed during the CFO. Unless competition workouts are known in advance or a much larger sample is accessible, allowing participants to select between Rx and scaled versions of each challenge may be unavoidable in future endeavors. Despite that limitation, our findings suggest that CFO workouts that provide an overload will stimulate a T response, whereas a lesser (or no) response may occur if modification is inappropriate. Regarding C, each workout elicited an elevated response, but longer duration workouts may elicit a greater response. When examined together, the TC ratio was mainly consistent throughout the CFO. Inconsistent responses in T, C, or TC may be indicative of inappropriate selection of workout difficulty, overstress, or both. Considering the potential influence of post-exercise concentrations in these hormones on performance, HIFT practitioners who continue their normal training should consider monitoring their changes during the CFO. Athletes and their coaches may find this useful for selecting between Rx and scaled workouts, and for modifying programming between weekly CFO workouts. However, these findings are inconclusive, possibly due to sample size limitations, the competitive environment, and time of day. Future research on these hypotheses is warranted.

## Figures and Tables

**Table 1:** Weekly event challenges for (male/female) competitors in the 2016 CrossFit^®^ Open.

Week	Scoring	Rx	Scaled
WK1	20-min AMRAP	25-ft overhead walking lunge (95/65 lbs.) 8 bar-over burpees 25-ft overhead walking lunge (95/65 lbs.) 8 chest-to-bar pull-ups	25-ft front rack walking lunge (45/35 lbs.) 8 burpees 25-ft front rack walking lunge (45/35 lbs.) 8 jumping chin-over-bar pull-ups
WK2	AMRAP or TTC *	25 toes-to-bar 50 double-unders Squat cleans: (135/85 lbs.) × 15 → (185/115 lbs.) × 13 → (225/145 lbs.) × 11 → 275/175 lbs.) × 9 → (315/205 lbs.) × 7	25 hanging knee raises 50 single-unders Squat cleans: (95/55 lbs.) × 15 → (115/75 lbs.) × 13 → (135/95 lbs.) × 11 → 155/115 lbs.) × 9 → (185/135 lbs.) × 7
WK3	7-min AMRAP	10 power snatches (75/55 lbs.) 3 bar muscle-ups	10 power snatches (45/35 lbs.) 5 jumping chest-to-bar pull-ups
WK4	13-min AMRAP	55 deadlifts (225/155 lbs.) 55 wall-ball shots (20/14 lbs. to 10/9 ft.) 55 calorie row 55 handstand push-ups	55 deadlifts (135/95 lbs.) 55 wall-ball shots (20/10 lbs. to 9/9 ft.) 55 calorie row 55 hand release push-ups
WK5	TTC	thrusters (95/65 lbs.) bar-over burpees 21-18-15-12-9-6-3 reps	thrusters (65/45 lbs.) bar-over burpees 21-18-15-12-9-6-3 reps

Note: AMRAP = As many repetitions as possible; Rx = As prescribed; TTC = Time to completion; * On WK2, competitors were allotted 4-min to complete the initial round of 90 repetitions (toes-to-bar, double-unders and squat cleans). Competitors progressed to the next round and received an additional 4 min if they completed all repetitions within the time cap, otherwise their workout was concluded. Progression continued this way until all 5 rounds were completed where TTC would distinguish performance.

**Table 2:** Competitor level and performance scores during each week of the 2016 CrossFit^®^ Open.

Participants	Week 1		Week 2			Week 3		Week 4		Week 5	
	Level	Repetitions	Level	Rounds	Repetitions	Level	Repetitions	Level	Repetitions	Level	Minutes
Women											
Participant #1	Scale	297	Scale	5	430	Scaled	143	Scale	278	Scale	13.78
Participant #2	Scale	216	Rx	1	79	Rx	10	Scale	165	Rx	18.38
Participant #3	Scale	135	Scale	4	348	Scale	49	Scale	121	Rx	26.98
Participant #4	Scale	218	Rx	2	20	Scale	108	Rx	165	Rx	19.93
Participant #5	Rx	669	Rx	2	167	Rx	58	Scale	217	Rx	15.25
Mean ± SD		307 ± 210		3 ± 2	209 ± 175		74 ± 52		189 ± 60		18.87 ± 5.15
Men											
Participant #6	Rx	685	Rx	2	165	Rx	75	Rx	178	-	-
Participant #7	Rx	791	Rx	2	173	Rx	90	Rx	199	Rx	13.47
Participant #8	Rx	681	Rx	2	172	Rx	71	Rx	184	Rx	14.47
Participant #9	Rx	681	Rx	2	175	Rx	75	Rx	182	Rx	18.38
Participant #10	Rx	720	Rx	2	126	Rx	89	Rx	193	-	-
Mean ± SD		719 ± 51		2 ± 0	162 ± 21		80 ± 9		187 ± 9	-	15.44 ± 2.60
All Participants		490 ± 265		2 ± 1	186 ± 120		77 ± 35		188 ± 41		17.58 ± 4.50

**Table 3:** Individual testosterone (pg·mL^−1^) responses during each week of the 2016 CrossFit^®^ Open.

Participants	Week 1				Week 2				Week 3				Week 4				Week 5			
	PRE	IP	30P	60P	PRE	IP	30P	60P	PRE	IP	30P	60P	PRE	IP	30P	60P	PRE	IP	30P	60P
Women																				
Participant #1	266	377	283	340	871	1222	563	686	202	1002	391	405	1479	4207	1250	704	186	661	387	375
Participant #2	180	591	280	159	232	660	361	273	148	333	297	239	59	225	98	38	21	41	42	29
Participant #3	67	127	99	84	79	110	112	73	72	71	95	84	89	81	117	89	69	87	102	53
Participant #4	59	396	132	110	89	197	66	44	46	296	62	102	78	361	160	55	36	256	121	78
Participant #5	90	261	166	103	74	148	169	114	85	172	176	161	99	135	157	44	78	174	100	100
Men																				
Participant #6	452	609	311	138	224	684	365	192	230	592	217	156	375	471	315	289	-	-	-	-
Participant #7	74	152	98	67	81	143	86	56	86	140	106	93	72	140	151	85	78	108	111	109
Participant #8	194	213	254	162	268	843	389	186	168	303	198	176	358	346	361	236	136	365	230	217
Participant #9	-	-	-	-	77	118	102	120	104	127	98	197	93	158	93	178	94	101	74	267
Participant #10	134	1685	358	205	95	750	203	224	154	826	466	418	290	1974	475	204	-	-	-	-

**Table 4:** Individual cortisol (ng·mL^−1^) responses during each week of the 2016 CrossFit^®^ Open.

Participants	Week 1				Week 2				Week 3				Week 4				Week 5			
	PRE	IP	30P	60P	PRE	IP	30P	60P	PRE	IP	30P	60P	PRE	IP	30P	60P	PRE	IP	30P	60P
Women																				
Participant #1	12.0	13.0	19.5	19.0	15.9	22.7	18.8	19.7	11.3	23.1	17.3	15.1	12.4	31.8	16.1	12.0	3.9	16.9	29.0	20.4
Participant #2	4.3	18.0	18.6	17.1	14.9	22.6	15.5	8.8	2.9	9.1	4.9	6.0	6.4	15.8	17.2	17.9	1.7	3.6	4.4	4.7
Participant #3	7.7	25.7	25.8	13.6	4.1	22.9	38.2	22.5	8.0	10.9	9.2	5.0	5.4	16.4	33.4	49.9	3.9	35.3	46.4	20.0
Participant #4	6.1	37.3	34.4	29.6	6.4	11.5	11.6	6.5	6.5	27.9	23.8	18.4	24.2	36.8	35.1	22.5	4.0	35.9	41.1	36.2
Participant #5	6.4	30.3	36.3	19.2	4.3	21.8	29.4	15.1	7.2	16.6	16.2	8.3	6.6	12.0	9.4	5.0	3.2	9.6	26.0	23.1
Men																				
Participant #6	6.8	15.7	10.4	6.5	8.8	11.4	9.5	4.8	3.6	11.8	6.9	5.2	5.4	20.0	10.9	4.1	-	-	-	-
Participant #7	3.3	21.0	14.8	11.3	4.7	13.8	8.5	5.0	8.8	14.4	8.4	4.5	1.5	10.9	8.1	5.3	0.8	12.4	14.4	9.3
Participant #8	12.2	18.8	23.0	24.8	19.0	26.9	24.5	7.8	14.9	19.4	19.4	14.2	18.5	20.4	26.0	16.2	6.1	13.3	30.7	21.1
Participant #9	-	-	-	-	6.3	8.9	8.8	9.7	6.1	11.4	8.5	7.6	5.6	16.9	18.8	13.4	2.9	3.4	5.4	3.6
Participant #10	28.8	54.4	39.7	34.4	12.4	35.6	25.5	17.7	11.5	29.0	25.7	17.8	23.8	46.2	28.2	22.8	-	-	-	-
